# Deletion of Dock10 in B Cells Results in Normal Development but a Mild Deficiency upon *In Vivo* and *In Vitro* Stimulations

**DOI:** 10.3389/fimmu.2017.00491

**Published:** 2017-05-01

**Authors:** Natalija Gerasimčik, Minghui He, Marisa A. P. Baptista, Eva Severinson, Lisa S. Westerberg

**Affiliations:** ^1^Department of Molecular Biosciences, The Wenner-Gren Institute, Stockholm University, Stockholm, Sweden; ^2^Department of Rheumatology and Inflammation Research, Institute of Medicine, University of Gothenburg, Gothenburg, Sweden; ^3^Department of Microbiology Tumor and Cell Biology, Karolinska Institutet, Stockholm, Sweden; ^4^Institute for Virology and Immunobiology, University of Würzburg, Würzburg, Germany

**Keywords:** B cells, Dock10, cytoskeleton, gene expression, humoral immune response

## Abstract

We sought to identify genes necessary to induce cytoskeletal change in B cells. Using gene expression microarray, we compared B cells stimulated with interleukin-4 (IL-4) and anti-CD40 antibodies that induce B cell spreading, cell motility, tight aggregates, and extensive microvilli with B cells stimulated with lipopolysaccharide that lack these cytoskeletal changes. We identified 84 genes with 10-fold or greater expression in anti-CD40 + IL-4 stimulated B cells, one of these encoded the guanine nucleotide exchange factor (GEF) dedicator of cytokinesis 10 (Dock10). IL-4 selectively induced Dock10 expression in B cells. Using lacZ expression to monitor Dock10 promoter activity, we found that Dock10 was expressed at all stages during B cell development. However, specific deletion of Dock10 in B cells was associated with a mild phenotype with normal B cell development and normal B cell spreading, polarization, motility, chemotaxis, aggregation, and Ig class switching. Dock10-deficient B cells showed lower proliferation in response to anti-CD40 and IL-4 stimulation. Moreover, the IgG response to soluble antigen *in vivo* was lower when Dock10 was specifically deleted in B cells. Together, we found that most B cell responses were intact in the absence of Dock10. However, specific deletion of Dock10 in B cells was associated with a mild reduction in B cell activation *in vitro* and *in vivo*.

## Introduction

B cells undergo cytoskeletal changes during their development, differentiation, and activation that are important for adhesion, motility, and cell-to-cell communication. The small Rho GTPases, Cdc42 and Rac1, are key regulators of cytoskeleton reorganization in B cells, where they control actin polymerization and microtubule dynamics (1–4). When overexpressed in B cells, constitutively active Cdc42 and Rac1 can induce filopodia and lamellipodia, respectively (5). Rho GTPases cycle between inactive GDP-bound and active GTP-bound forms. Members of the family of guanine nucleotide exchange factors (GEFs) regulate the activity of Rho GTPases by exchanging the binding of GDP for that of GTP (6, 7).

Dock10, also known as Zizimin3, is a GEF that is a member of the Dock-D subfamily, as are Dock9 (Zizimin1) and Dock11 (Zizimin2) (6, 8). Proteins of this subfamily contain a pleckstrin domain that can specifically activate Cdc42 and other closely related GTPases (9). Yelo et al. (10) and Alcaraz-Garcia et al. (11) have shown that Dock10 is encoded by a gene that is induced by interleukin-4 (IL-4) in chronic lymphocytic leukemia (CLL) cells and in human peripheral blood B lymphocytes but not T lymphocytes (10, 11). The rapid induction of Dock10 after IL-4 stimulation in CLL and normal peripheral blood B cells suggested that Dock10 is important for IL-4-induced B cell activation (10). Dock10 is a GEF for Cdc42 and Rac1, and it plays an important role in amoeboid migration as well as dendritic and filopodia formation (12–14). Amoeboid migration is a mode of movement used by non-adherent or rounded cells, which do not form focal adhesions, whereas adherent cells use mesenchymal movement (15). Silencing of Dock10 expression leads to partial suppression of migration, and the simultaneous knock down of Dock10 and Rac1 suppresses migration completely, resulting in a decreased invasion of melanoma cells (12).

We have previously shown that IL-4 and anti-CD40 induce striking morphological changes in B cells, such as B cell spreading and the formation of large aggregates with dense microvilli in cell-to-cell contacts (16, 17). Moreover, B cell spreading is largely inhibited in B cells devoid of Cdc42 (4). In addition, IL-4 can change the activation status of Cdc42 (5). The B cell cytoskeletal changes depend partly on the activity of the Wiskott–Aldrich syndrome protein, a downstream effector of Cdc42 (5, 18). Since IL-4-stimulated cytoskeletal changes are induced 6–12 h after activation (16), we suspected that these processes need *de novo* gene transcription. To identify and test the function of genes that regulate cytoskeletal changes in B cells, we used microarray analysis and compared the mRNA expression profiles of B cells stimulated with anti-CD40 + IL-4 with those of LPS-stimulated B cells. We found that Dock10 is selectively induced by IL-4 stimulation. Conditional depletion of Dock10 in B cells revealed a mild phenotype, and the major observable change was a lower DNA synthesis induced by IL-4 and anti-CD40 and a lower IgG response to a soluble T cell-dependent (TD) antigen.

## Materials and Methods

### Mice and Immunizations

Dock10 (B6NTac; B6N-Dock10^tm1a(EUCOMM)Hmgu^/Ieg) mutant mice were purchased from EMMA (European Mouse Mutant Archive, Helmholtz Zentrum München—German Research Center for Environmental Health GmbH) (19, 20). Dock10 mutant mice were constructed so that exon 4 of *dock10* was flanked by loxP sites to enable its conditional deletion in Cre-expressing mice. In addition, intron 3 contains the gene encoding lacZ, flanked by FRT sites (21) (see Figure 2A). We first crossed Dock10 mutant mice with Flp-expressing mice, to yield Dock^fl^ mice (Figure 3A). They were thereafter crossed with two different Cre-expressing mice: Mb1-Cre-ERT2 mice, which were a gift from Michael Reth, University of Freiburg (22), or CD23Cre mice, which were a gift from Meinrad Busslinger, Vienna Biocenter (23). These two crossings enabled deletion of Dock10 in most lineages of B cells, from pro-B cells to activated B cells or mature B cells, and we call them Dock10^fl/fl^Mb1Cre-ERT2 and Dock10^fl/fl^CD23Cre, respectively. In addition, we crossed the Dock10 mutant mice with the Cre-expressing mice Mb1-Cre-ERT2 or CD21Cre mice (24) (see Figure 2A). In the CD21Cre mice, Cre will be expressed in mature B cells. This allowed lacZ expression to be controlled by the Dock10 promoter, and thus these mice can be used to determine which populations of cells express Dock10. We call these strains Dock10^lacZ/+^Mb1Cre-ERT2 and Dock10^lacZ/+^CD21Cre, respectively. All strains were on the C57Bl/6 background, and further breedings were carried out using this strain. To achieve Dock10 deletion in the Mb1-Cre-ERT2 combination, mice were given tamoxifen (5 µg in 50 µl) by gavage for 5 days in a row. For *in vitro* cultures, mice were sacrificed on day 3 after the final tamoxifen treatment. Mice were immunized with either sheep erythrocytes (SRBC) or trinitrophenyl (TNP)-SRBC on day 4 after the final tamoxifen treatment. The erythrocytes were diluted to a 10% mixture from packed cells, and 0.2 ml were injected i.p. 4-hydroxy-3-nitrophenylacetyl coupled to keyhole limpet hemocyanin (NP-KLH) (100 μg/mouse in alum or 20 μg/mouse in PBS for recall) were injected i.p. Mice were bled from the tail or by retro-orbital bleeding in anesthetized mice. Mice were used between 6 weeks and 6 months, except for the long-term immunization experiments, in which the mice were 8 months when sacrificed.

### Cell Culture

B cells were purified from spleens by negative selection, using a mouse B cell enrichment kit (Stem Cell Technologies), followed by separation in a Percoll gradient (GE Healthcare). For analysis of Ig class switching, spleen B cells were enriched by sequential incubation with antibodies to CD4, CD8, CD90.2, and CD11b (BD Biosciences or eBioscience) and low-tox rabbit complement (Cedarlane), with a wash in between, followed by Percoll separation. B cells were cultured at 2–4 × 10^5^ cells/ml, as described previously (5). Monoclonal rat-anti-mouse CD40 mAbs (1C10) were purified as described previously (25) and were used at 10–20 µg/ml. IL-2, IL-4, IL-5, IL-21, BAFF, and April were purchased from Peprotech. IL-4 was used at 2–16 ng/ml, IL-2 and IL-5 at 5 ng/ml, IL-21 at 20 ng/ml, and BAFF and April at 100 ng/ml. Lipopolysaccharide O55:B5, purified by phenol extraction (LPS), was purchased from Sigma-Aldrich and was used at 10 µg/ml. F(ab′)_2_ goat-anti-mouse IgM (Jackson ImmunoResearch) was used at 2 µg/ml. For spreading assays, cells were first stimulated with LPS or anti-CD40 + IL-4 mAb for 1 day and then transferred to wells with glass coverslips coated with 50 µg/ml anti-CD44 (BD Biosciences) and then cultured for 2–24 additional hours. Spread cells were defined as cells with at least one protrusion longer than one cell diameter. For polarization, spleen B cells were stimulated with 5 ng/ml IL-4 for 18 h and thereafter washed and fixed. Polarized cells are defined as non-round cells, which are tapered and have uropods.

All mouse cell lines (Pre-B Lymphoblast 70Z/3; B Lymphocytes: WEHI-231, L10A6.2, BCL1 clone 5B1b, A20.3, M12.4.1; and Plasmacytoma S194/5.XXO) were cultured in complete RPMI 1640 medium (Gibco), supplemented with 10–15% heat-inactivated fetal calf serum (FCS).

### Microarray

Purified B cells from three mice per group were activated in separate by anti-CD40 + IL-4 or by LPS for 2 days. For non-stimulated spleen cells, two spleens per group were pooled, and a total of three groups were analyzed. Total RNA was extracted and amplified to digoxigenin (DIG)-labeled cRNA using the Applied Biosystems Chemiluminescent NanoAmp™ RT-IVT Labeling Kit. The labeled DIG-cRNA (10 µg per microarray) was incubated in each microarray hybridization chamber at 55°C for 16 h. The microarrays were washed. Features that retained bound DIG-labeled cRNA were visualized using the Applied Biosystems Chemiluminescence Detection Kit. Anti-DIG alkaline phosphatase was used to hydrolyze a chemiluminescence substrate to generate light at 458 nm, which was then detected by the Applied Biosystems 1700 Chemiluninescent Microarray Analyzer. The Mouse Genome Survey Microarray v.2.0 (Applied Biosystems) Microarrays were preprocessed using the R bioconductor package (http://www.r-project.org). Probes with unusual signal patterns were flagged by the scanner and signal strength set to NA (not a number). Missing intensity values were imputed using the nearest neighbor method. The intensity matrix was normalized by RMA normalization. All microarray data have been deposited in NCBI’s Gene Expression Omnibus database with accession number GSE96813 and can be accessed at https://www.ncbi.nlm.nih.gov/geo/query/acc.cgi?acc=GSE96813. For the heatmaps, gene expression microarray data were analyzed using Qlucore Omics Explorer 3.2 (Qlucore AB, Sweden).

### Real-time Reverse Transcriptase Polymerase Chain Reaction (RT-PCR)

Non-stimulated or stimulated B cells were harvested as indicated in the figure legends. Total cellular RNA was isolated using the GenElute Mammalian Total RNA Miniprep Kit (Sigma). For cDNA synthesis, 2 µg of total RNA was used. RT-PCR was carried out using TaqMan Master Mix and 20× TaqMan Gene Expression Assays [Dock8: Mm00472344_m1, Dock10: Mm00614273_m1, Dock11: Mm01297590_m1, GAPDH: Mm03302249_m1, and Mb-1 (CD79a): Mm00432423_m1, Applied Biosystems]. Samples were run in Thermo Fast 96 PCR plates (96-well ABgene PCR plates, Thermo Scientific) with the ABI PRISM 7000 Sequence Detection System (Applied Biosystems), using an absolute quantification in ABI PRISM 7000 SDS Software. The results from primary activated B cells were normalized to Mb-1 and non-stimulated B cells; those of cell lines were normalized to GAPDH and 70Z/3. The degree of increase or decrease was calculated based on the amplification efficiency for each gene. The degree of induction by IL-4 for cell lines was normalized to non-stimulated cell lines and the degree of increase was calculated using the amplification efficiency of Dock10.

### Western Blot

Non-stimulated and stimulated B cells were harvested as described in the figure legends. Twenty micrograms of total cell extracts were separated by SDS-PAGE and analyzed by western blot, using polyclonal rabbit anti-human Dock10 (Abcam) antibodies and polyclonal rabbit anti-α-tubulin or monoclonal mouse anti-α-tubulin (both from Abcam) antibodies, with HRP-conjugated swine anti-rabbit Ig (Dako) or rabbit anti-mouse Ig-HRP (Dako). The intensities of the bands are specified relative to that of α-tubulin.

### Flow Cytometry

Single-cell suspensions were labeled with fluorescently conjugated anti-mouse antibodies, including anti-B220, -CD21, -CD23, -CD24, -CD38, -CD43, -CD73, -CD83, -CD93, -CD95 (Fas), -CD138 (Syndecan), -GL7, -CXCR4, -IgM, -IgD, and -I-A/I-E (MHC class II) (BD Biosciences, eBiosciences, Biolegend). A LIVE/DEAD Fixable Dead Cell Stain Kit (Molecular Probes) was used to identify viable cells. Different B cell populations were defined as following: peritoneal B cells: B1 (CD3^−^B220^+^CD23^−^) and B2 (CD3^−^B220^+^CD23^+^); bone marrow fractions (26): fr.A or pre-pro-B cells (CD43^+^B220^lo^CD24^−^), fr.B + C or pro-B cells (CD43^+^B220^lo^CD24^+^), fr.D or pre-B cells (CD43^−^B220^+^IgM^−^IgD^−^), fr.E or new-B cells (CD43^−^B220^+^IgM^+^IgD^−^), fr.F or mature B cells (CD43^−^B220^+^IgM^+^IgD^+^); splenic B cells: T1 (B220^+^CD23^−^IgM^+^CD21^−/lo^), T2-marginal zone precursor (B220^+^CD23^+^IgM^+/hi^CD21^+/hi^), FOB (B220^+^CD23^+^IgM^+/lo^CD21^+/lo^), MZB (B220^+^CD23^−^IgM^+^CD21^hi^), naïve B cells (B220^+^CD38^+^GL7^−^CD73^−^), memory B cells (B220^+^CD38^+^GL7^−^CD73^+^), germinal center (GC) B cells (B220^+^CD38^−^GL7^+^CD95^+^), GC light zone (LZ) and dark zone (DZ) B cells (B220^+^CD38^−^GL7^+^CD95^+^CD83^+^/^−^CXCR4^+^/^−^), plasmablasts (PB) (B220^+^CD138^+^), and plasma cells (PC) (B220^−^CD138^+^). Fluorescence minus one controls were used when defining different populations of B cells, and isotype controls were used in switching experiments. For the Ig class switching, single-cell suspensions were fixed with formaldehyde, permeabilized with saponin, and labeled with biotinylated antibodies to IgM, IgG1, IgE, IgG2b, IgG3, or isotype controls (BD Biosciences). Streptavidin–FITC was used as a second step. The expressions of Dock10 in different B cell populations were analyzed by detecting β-galactosidase (LacZ) expression, using FACS-Gal analysis (27). The method is based on the detection of LacZ^+^ cells with fluorescein di(β-d-galactopyranoside) (FDG, Sigma), a fluorogenic substrate. LacZ cleaves FDG, and a fluorescent product (FITC) that can be measured by flow cytometry is released. Data were acquired on FACSCalibur, FACSVerse, or LSR Fortessa (Becton Dickenson). Analyses were made using FlowJo (TreeStar Inc.).

### Live Cell Imaging

Cell polarization, spreading, and aggregation were analyzed using a ZEISS Axiovert 200M Cell Observer and SlideBook 5.0 Software. A HAL100 halogen lamp was used for bright-field imaging. Images were taken with a digital camera AxioCam MRc. EC-Plan-Neofluar 10×/0.3 Ph1, or EC-Plan-Neofluar 40×/0.75 Ph2 lenses were used. Experiments were performed at 37°C in medium supplemented with 10 mM HEPES buffer to keep constant pH.

### Immunofluorescence Microscopy

Stimulated B cells on glass coverslips were fixed, washed, permeabilized, treated with anti-mouse CD16/CD32, and then stained with fluorescently conjugated phalloidin, Hoechst 33258 (both from Sigma-Aldrich), rat-anti-mouse α-tubulin (Abcam), and fluorescently conjugated donkey-anti-rat Ig (minimal cross-reactivity to mouse Ig, Jackson ImmunoResearch). Cells were observed, and images were captured using a Leica DMLB Fluorescent Microscope, HCX PL APO 63×/1.32 oil lens, DC350F CCD camera, and IM500 Software (Leica Microsystems).

### Transwell Migration Assay

*In vitro* migration of B cells to CXCL12 (SDF-1) was assessed, as previously described (18). In brief, 2 × 10^5^ splenic B cells were added to the upper chamber of Transwell cultures (Corning Inc.), and 100 ng/ml CXCL12 were added to the lower chamber. Migration of B cells to the lower chamber was analyzed by flow cytometry after 2 h of incubation at 37°C and expressed as percentage of total B cells. Each condition was set up in triplicates. *In vitro* migration of B cells to IL-4 was assessed similarly as to CXCL12. In brief, 2 × 10^5^ splenic B cells were added to the upper chamber of Transwell cultures, and 5 ng/ml IL-4 were added to the lower chamber. Migration of B cells to the lower chamber was analyzed by flow cytometry after 18 h of incubation at 37°C and expressed as percentage of total B cells. Each condition was set up in triplicates.

### Genotyping

DNA for genotyping was isolated from tails of 3–4 weeks old mice. All primers were purchased from https://biomers.net GmbH. Dock10 F1R3 primers were used to detect the presence of the transgene (product length 558 bp); Dock10 F1R4 to detect knockout (865 bp); and Dock10 F5R5 to detect WT (417 bp). Dock10 F1: 5′-ATAAAATCAAAGCTTAGGGGAA-3′, Dock10 R3: 5′-GCTTTTGTTACTGCCGCCTA-3′, Dock10 R4: 5′-CAAACTTGACCCAGTGCAT-3′, Dock10 F5: 5′-TATTAAAGGAGCAGAGGGAACAG-3′, Dock10 R5: 5′-ATTTACAAGCCTGAAATGGAC-3′. Tail biopsies and typing were always performed twice, once after weaning and once when the mice were sacrificed.

### DNA Synthesis

Purified B cells were cultured at 10^6^ cells/well in 0.2 ml in 96-well cultures in complete RPMI 1640 plus 10% FCS. [^3^H]thymidine (5 μCi/culture, 20 Ci/mmol, PerkinElmer) was added 17 h before harvesting. Cultures were harvested, and incorporated radioactivity was measured using a Wallac microplate scintillation counter (Wallac Oy, Turku, Finland).

### ELISA

Diluted serum samples were added to 96-well plates, preincubated with TNP-BSA or NP-BSA, the latter with 8 or 25 hapten molecules per molecule of BSA, and thereafter washed and incubated with binding buffer. Plates were washed after serum addition, and alkaline phosphatase conjugated antibodies to IgM or IgG were added and measured by ELISA. Standard monoclonal anti-TNP antibodies were used for the TNP responses. For the NP responses, we used pooled antisera in different dilutions as standard, to be able to calculate relative concentrations of the tested sera. An arbitrary value of 100 U corresponded to pooled antisera from week 1 diluted 1:100 for IgM and pooled antisera from week 2 diluted 1:800 for IgG.

### Statistical Analysis

Statistically significant differences between groups were assessed by Student’s *t-*test for paired or unpaired analysis and ANOVA with *post hoc* Sidak test for multiple comparisons. Differences were considered significant when *p* ≤ 0.05.

## Results

### Identification of Genes Expressed in B Cells Stimulated by Anti-CD40 and IL-4

To identify and test the functions of genes that regulate cytoskeletal changes in B cells, we compared mRNA expression profiles by microarray analysis of B cells activated with two different stimuli. IL-4 in combination with antibodies to CD40 (anti-CD40) mimics TD stimuli and induces cytoskeletal changes that include B cell spreading, and the formation of large dense aggregates with microvilli in cell-to-cell contacts. The gene expression profile of anti-CD40 + IL-4 stimulated B cells was compared to that of LPS-stimulated B cells, which remain round and form only loose aggregates. The profile showed that 5,000 genes of the total of 30,000 were significantly differently expressed comparing these two conditions. Of these, 2,367 genes were expressed more than 2-fold higher in anti-CD40 + IL-4 than in LPS-activated cells (Data Sheet S1 in Supplementary Material), while 84 genes showed 10-fold or greater expression (Data Sheet S1 and Figure S1 in Supplementary Material). In addition, 161 genes were at least twofold upregulated in LPS versus anti-CD40 + IL-4-stimulated cells (Data Sheet S1 in Supplementary Material). The gene that encodes Dock10 was expressed almost 14 times higher in cultures activated with anti-CD40 + IL-4 as compared to those activated by LPS (Data Sheet S1 in Supplementary Material). This caught our attention, since Dock10 is a GEF for Cdc42 and a candidate for inducing cytoskeletal changes in B cells.

### IL-4 Induces Upregulation of Dock10 Expression in Murine Primary B Cells and Malignant B Cell Lines

**Figure 1:**
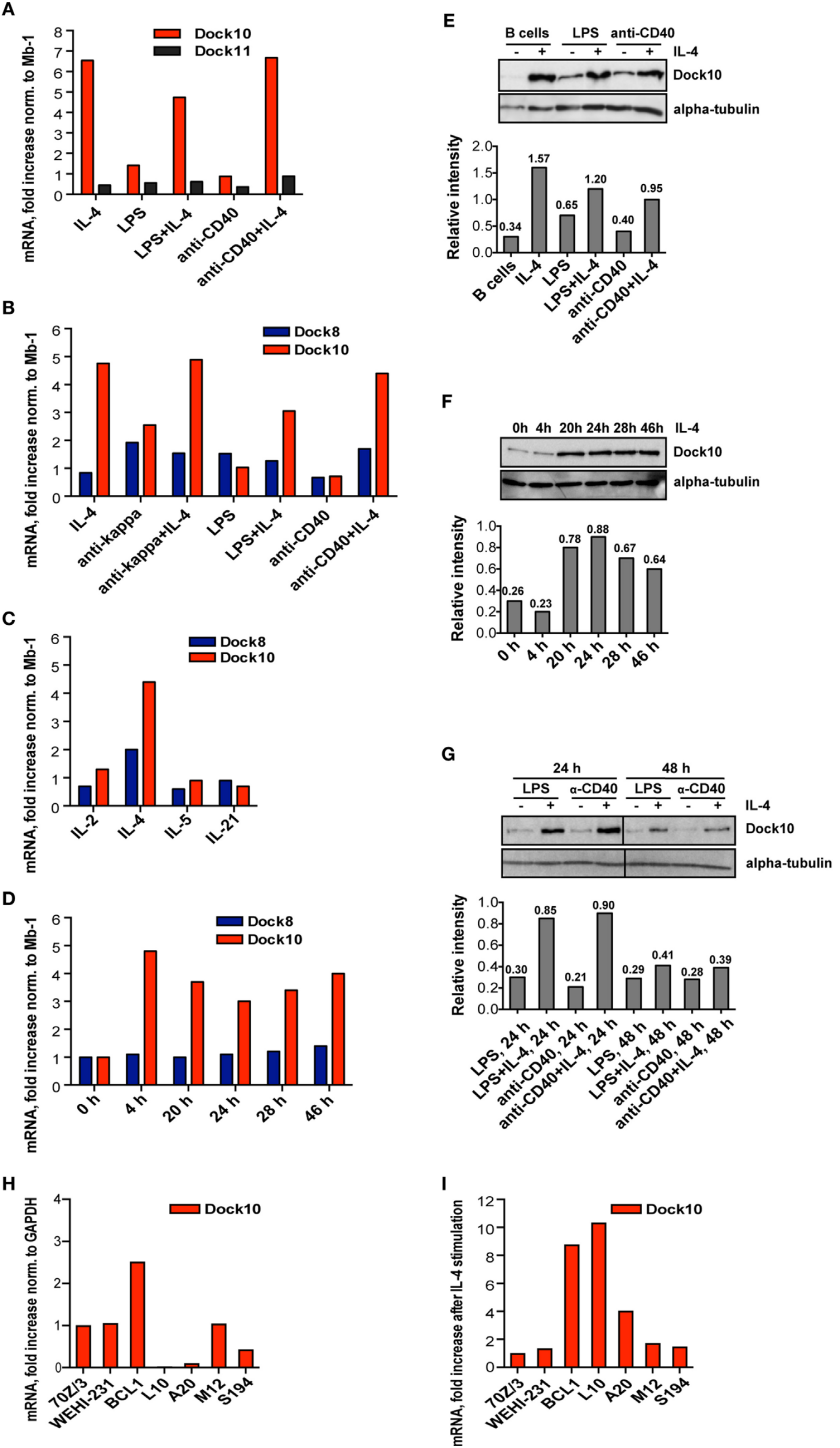
**Interleukin-4 (IL-4) induces upregulation of Dock10 expression in murine primary B cells and malignant B cell lines**. **(A–D,H,I)** mRNA expression of various genes by real-time reverse transcriptase polymerase chain reaction. The values represent the mean and SD of three biological replicates. **(A–D)** Values are compared to the reference gene Mb-1, which is set to 1. **(H,I)** Values are compared to the reference gene GAPDH, which is set to 1. **(E–G)** Protein expression as analyzed by Western blot. The relative intensities are specified relative to that of α-tubulin. **(A,B)** mRNA expression of **(A)** Dock10 and Dock 11 and **(B,C)** Dock8 and Dock10 after 24 h stimulation of spleen B cells. Values are shown as fold increase normalized to non-stimulated spleen B cells. **(D)** Kinetics of Dock10 mRNA after IL-4 stimulation. Cells were harvested at the indicated time points. **(E)** Dock10 protein expression in spleen B cells upon stimulation for 24 h. **(F)** Kinetics of Dock10 protein expression in spleen B cells stimulated for 0–46 h with IL-4. **(G)** Dock10 protein expression in spleen B cells after stimulation for 24 or 48 h. **(H)** Dock10 expression in mouse B cell lines. The results are shown as fold expression of Dock10, normalized to the pre-B cell line 70Z/3 set to 1. **(I)** Fold increase of Dock10 expression in mouse B cells incubated for 24 h with IL-4. The data are representative of three experiments **(A–D)**, four experiments **(E)**, and two experiments **(F–I)** with similar results.

To understand how the expression of Dock10 is regulated in B cells, we added different stimuli to murine B cells and examined the effects by RT-PCR. IL-4 alone or in combination with LPS, anti-CD40, or anti-Igκ, but not other stimuli, induced strong Dock10 expression (Figures 1A,B). Dock11, a homolog of Dock10 belonging to the same subfamily, or Dock8, a GEF that belongs to another subfamily (6), were both expressed in B cells but were not reproducibly upregulated by any of the stimuli tested (Figures 1A,B). When comparing the expression levels of the close homologs Dock10 and Dock11 relative to that of Mb-1, we found that Dock11 was expressed at twofold higher levels than Dock10 in non-activated B cells (2.0 ± 0.4, mean and SD of three experiments). In IL-4 stimulated B cells, the situation was reversed; Dock10 was expressed almost four times higher than Dock11 relative to that of Mb-1 (3.9 ± 1.99, mean and SD of three experiments). However, Dock9, another member of the same family as Dock10 and Dock11, was expressed at very low levels in B cells (data not shown). None of the cytokines IL-2, IL-5, or IL-21 were able to influence Dock10 expression (Figure 1C). IL-4 induced the highest Dock10 mRNA 4 h after stimulation, and expression remained high up to 46 h after stimulation (Figure 1D). Dock10 protein expression followed a similar pattern, being maximally induced at 24 h and remaining elevated during 48 h (Figures 1E–G).

The Dock10 protein is expressed in human CLL cells and was further upregulated by IL-4 (10). We wished to investigate if murine malignant B cell lines were different in Dock10 gene expression and to relate this to the differentiation stages of various cell lines. We tested seven B cell lines, one of which was in a pre-B cell stage (70Z/3) and was used as a control. Other cell lines corresponded to mature B lymphocytes, expressing either IgM (BCL1, WEHI-231, and L10) or IgG (A20 and M12). The plasmacytoma S194 secretes IgA antibodies. When compared to 70Z/3, the BCL1 cell line had higher expression of Dock10 (Figure 1H), whereas L10, A20, and S194 had the lowest expression. Upon addition of IL-4, Dock10 expression was increased in BCL1, L10, and A20 cell lines, but not in the others (Figure 1I). In summary, Dock10 expression is strongly induced by IL-4 in both primary B cells and B cell lines (L10, BCL1, and A20) that correspond to mature B cells. These data suggest that the BCL1 tumor cell line could provide a good tool to investigate if Dock10 provides a selective advantage for B cell leukemias. However, since we have only investigated Dock10 mRNA expression, there is a possibility that Dock10 protein levels may be differently regulated.

### Dock10 Is Expressed in B Cells of All Differentiation Stages

**Figure 2:**
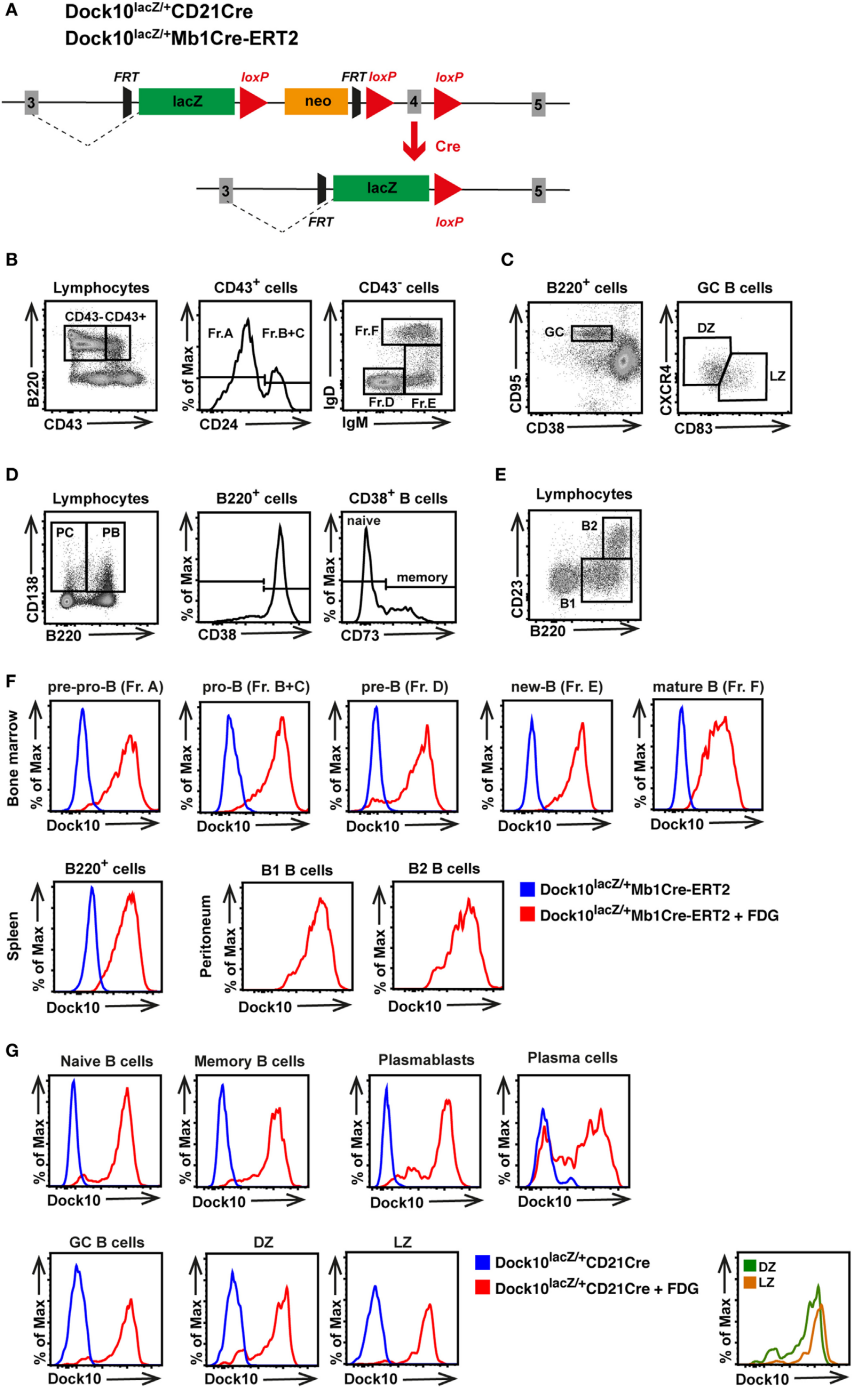
**Dock10 is expressed in B cells of all differentiation stages**. **(A)** Generation of the Dock10-LacZ reporter mouse for B cells. **(B–E)** Flow cytometry gating strategy for **(B)** bone marrow B cells using Hardy classification for fraction (Fr) A–F, **(C)** germinal center (GC) B cells including GC light zone (LZ) and dark zone (DZ) B cells, **(D)** plasmablasts (PB), plasma cells (PC), and memory B cells, **(E)** peritoneal cavity B1 and B2 B cells, **(F)** Dock10-lacZ expression in the indicated organ of Dock10^lacZ/+^Mb1Cre-ERT2 mice with or without the FDG substrate for lacZ detection. **(G)** Dock10-lacZ expression in Dock10^lacZ/+^CD21Cre mice with or without the FDG substrate for lacZ detection. Mice were immunized with SRBC and B cell subpopulations analyzed on day 7; FDG, fluorescein di-β-d-galactopyranoside. **(F,G)** The experiments were performed with cells from one mouse each. Another experiment with one mouse per group was performed with similar results.

To examine Dock10 expression in different B cell subpopulations during development, Dock10^lacZ/+^ mice were used that express LacZ under the Dock10 promoter (Figure 2A). Dock10^lacZ/+^ mice were crossed with Mb1Cre-ERT2 or CD21Cre mice, which results in replacement of exon 4 with the lacZ gene, specifically in B cells in most differentiation stages upon tamoxifen administration or in mature B cells, respectively. Using this approach, Dock10-lacZ expression in different B cell subpopulations was examined by staining for lacZ using the fluorogenic substrate of galactosidase (FDG) and flow cytometry analysis (Figures 2B–G). Only background staining was observed in cells not treated with FDG (blue color in Figures 2F,G). When examining Dock10^lacZ/+^Mb1Cre-ERT2 mice, we found that Dock10 was expressed in all subpopulations, from pro-B cells to mature B cells, in the bone marrow (red color, Figure 2F). Dock10 was also expressed in spleen B cells, as well as in B1 and B2 cells of the peritoneum (Figure 2F). To examine the expression of Dock10 upon antigen challenge, Dock10^lacZ/+^CD21Cre mice were immunized with TNP-conjugated sheep red blood cells (TNP-SRBC). The majority of the different B cell subpopulations expressed Dock10 in the spleen of immunized mice (Figure 2G). The lower Dock10-lacZ expression in the GC DZ B cells, PB, and PC is most likely due to a downregulation of Dock10 expression as B cells differentiate (Figure 2G).

### Specific Deletion of Dock10 in B Cells Reveals a Normal Phenotype in Subpopulations of B Cells and B Cell Morphology

**Figure 3:**
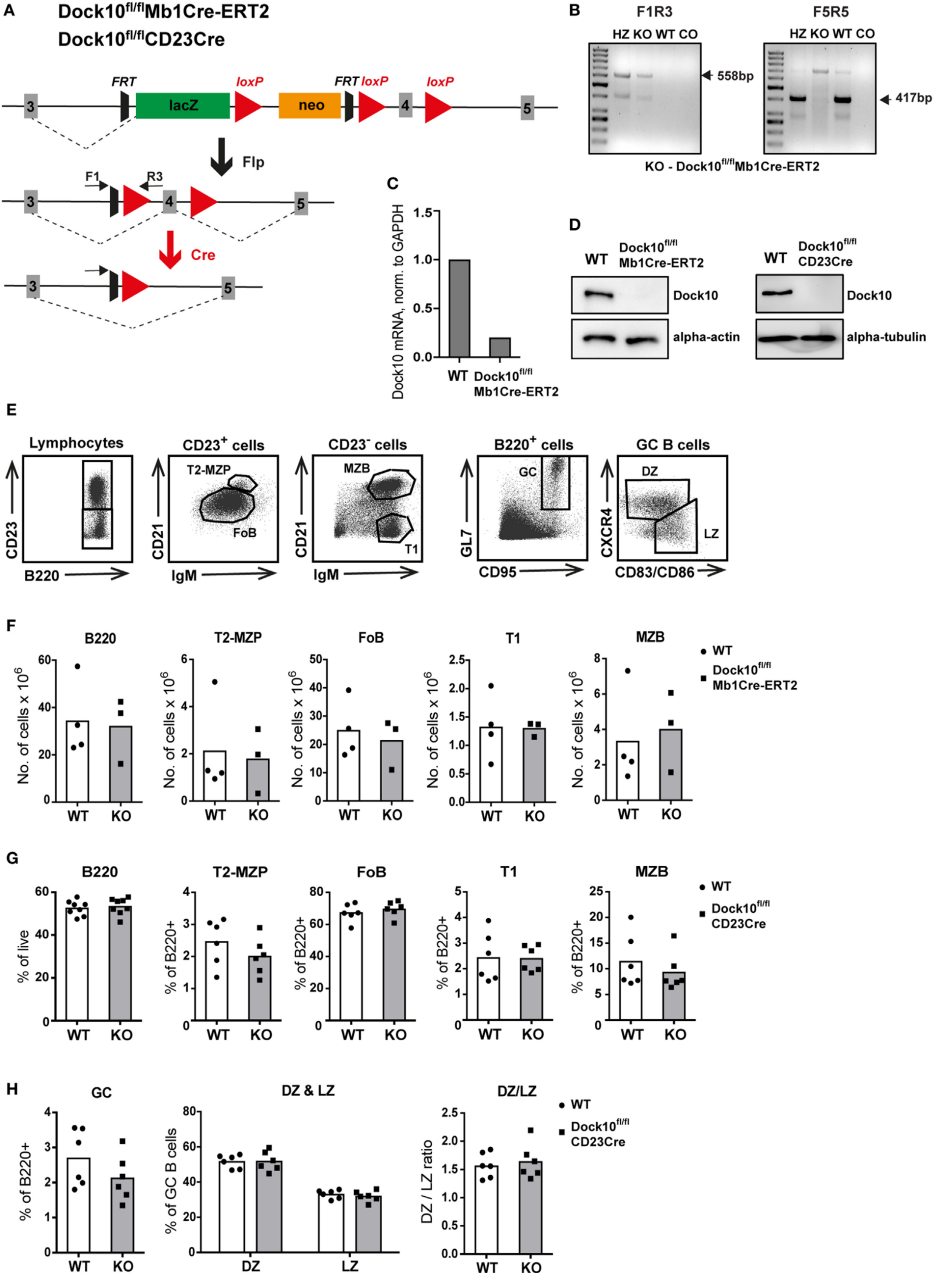
**Specific deletion of Dock10 in B cells results in normal B cell development**. **(A)** Generation of the conditional allele and knockout allele for B cell specific deletion of Dock10. The location of F1 and R3 primers are indicated. Both correspond to regions upstream of exon 4, flanking the FRT site and R3 partly covering the most upstream loxP site. **(B)** PCR analysis of tail DNA as a typing example, showing Dock10^fl/fl^ (KO), Dock10^fl/+^ (HZ), wild-type (WT), and no template control (CO) with primer combination F1R3 to detect the transgene (left) and with primer combination F5R5 (right) to detect the WT Dock10 gene. The F5 primer covers a region, which is not present in the transgene, just upstream of exon 4, whereas R5 partly covers the 5′ region of exon 4, and thus there will be no F5R5 product in mice homozygous for the transgene (Dock10^fl/fl^). The sizes of the expected bands are indicated. Sometimes with old primers, bands of other sizes might appear. **(C)** Real-time reverse transcriptase polymerase chain reaction analysis of the expression of Dock10 mRNA in purified splenic B cells from WT and Dock10^fl/ fl^Mb1Cre-ERT2 mice, stimulated for 2 days with anti-CD40 + IL-4, normalized to WT and GAPDH. **(D)** Expression of Dock10 protein in splenic B cells from WT and Dock10^fl/fl^Mb1Cre-ERT2 mice, stimulated for 2 days with anti-CD40 + IL-4 (left) or B cells from WT and Dock10^fl/fl^CD23Cre mice, stimulated for 3 days with anti-CD40 + IL-4 (right). **(E)** Gating strategy for indicated B cell subpopulations. **(F–H)** Flow cytometry analysis of B cell subpopulations; **(F,G)** transitional (T1), follicular B cells (FO), T2-marginal zone precursor (T2-MZP), marginal zone (MZ) B cells, and **(H)** germinal center (GC) B cells including GC light zone (LZ) and dark zone (DZ) B cells. **(F)** Analysis of naïve Dock10^fl/fl^Mb1Cre-ERT2; *n* = 3, WT; *n* = 4. **(F,G)** Analysis of Dock10^fl/fl^CD23Cre mice on day 9 after secondary immunization with NP-KLH; *n* = 6–8 per group.

To define the role of Dock10 for B cell development and function, we used a conditionally targeted Dock10 allele (Dock10^fl^) and bred these mice with Mb1-Cre-ERT2 (Mb1Cre-ERT2) mice to delete Dock10 specifically in most subpopulations of B cells upon tamoxifen administration (Figure 3A). Examples of typing for the transgene Dock10^fl/fl^, Dock10^fl/+^, or WT gene, using tail DNA is shown in Figure 3B. As shown in Figure 3C, expression of *dock10* mRNA was reduced fivefold in B cells of Dock10^fl/ fl^Mb1Cre-ERT2 mice. In this experiment, the primers spanned the most downstream exons 51 and 52, indicating that the entire *dock10* gene was expressed at much reduced levels. Floxed deletion of exon 4 is expected to introduce a premature stop codon by changing the reading frame and abolishing Dock10 expression, and we detected no Dock10 protein expression in Dock10^fl/fl^Mb1Cre-ERT2 B cells stimulated with anti-CD40 + IL-4 for 2 days (Figure 3D). We also bred mice with the Dock10^fl^ allele with CD23Cre-expressing mice, in order to study long-term antibody responses (Figures 6C–I). Also in this combination, there was no detectable expression of Dock10 in activated B cells (Figure 3D). The anti-Dock10 antibodies that were used in the western blot were made against a synthetic peptide that partly corresponded to exon 4 in the Dock10 gene. Unfortunately, no other suitable antibody was available to us. Thus, we cannot exclude that Dock10^fl/fl^Mb1Cre-ERT2 and Dock10^fl/fl^CD23Cre B cells express a low level of a truncated form of Dock10, lacking exon 4. By administration of tamoxifen and analysis of Dock10^fl/fl^Mb1Cre-ERT2 B cells on day 8, Dock10-deficient B cells did not significantly differ in numbers of various subpopulations in spleen as compared to control Cre^−^ B cells (Figures 3E,F). In addition, using Dock10^fl/fl^CD23Cre mice, the percentage of different B cell populations did not differ significantly from controls (Figures 3G,H).

### Dock10 Is Not Required for B Cell Spreading, Polarization, Motility, Migration, Chemotaxis, or Aggregation

**Figure 4:**
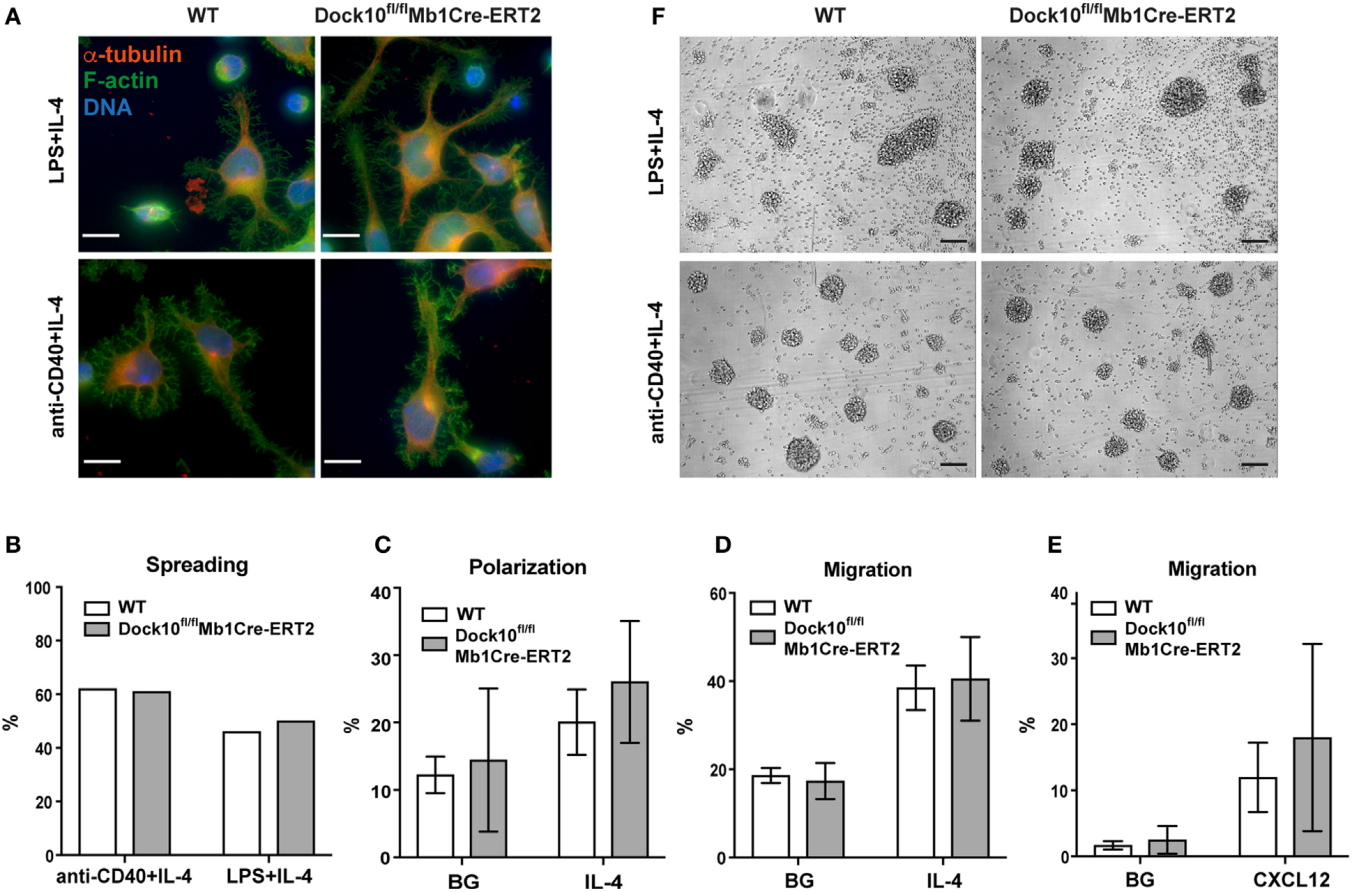
**Dock10 is not required for B cell spreading, polarization, motility, migration, chemotaxis, or aggregation**. **(A)** Spreading of B cells derived from wildtype (WT) or Dock10^fl/fl^Mb1Cre-ERT2 mice stimulated with LPS + IL-4 or anti-CD40 + IL-4 and stained for F-actin with Phalloidin and α-tubulin. DNA was stained with Hoechst 33258. Scale bars, 10 µm. Representative results from two independent experiments are shown. **(B)** Quantification of spreading of WT or Dock10^fl/fl^Mb1Cre-ERT2 B cells stimulated with LPS + IL-4 or anti-CD40 + IL-4. Around 700–900 cells were counted per mouse per stimuli. The experiment was repeated once with similar results. **(A,B)** Cells were cultured for 20 h after being transferred to anti-CD44 coated glass coverslips. **(C)** Polarization analysis of WT or Dock10^fl/fl^Mb1Cre-ERT2 B cells stimulated with IL-4. Three independent experiments with one mouse per group were performed. **(D)** Migration analysis of WT or Dock10^fl/fl^Mb1Cre-ERT2 B cells stimulated with IL-4, three independent experiments with one mouse per group were performed. **(E)** Chemotaxis toward CXCL12, four independent experiments with one mouse per group was performed. **(F)** B cell aggregation upon stimulation with LPS + IL-4 or anti-CD40 + IL-4 for 50 h. Bright-field microscopy images, scale bars, 100 µm. One additional experiment was performed with similar results. IL-4, interleukin-4; BG, background.

We recently found that deletion of Cdc42 severely compromised the capacity of B cells to spread on antibody-coated monolayers (4). Furthermore, we have been able to associate spreading with a particular type of movement of B cells, when they are cultured in liquid media. The purpose of this study was to find GEFs that regulate Cdc42-induced morphological changes, such as spreading. WT or Dock10-deficient B cells were activated with anti-CD40 + IL-4 for 1 day and then transferred to anti-CD44 coated glass surfaces and cultured for an additional day, after which the percentage of spread cells was determined. Antibodies to diverse B cell surface receptors are capable of mediating cell spreading including antibodies to CD44, CD23, VLA-4, and ICAM-1 (16). CD44 was chosen since CD44 interacts with cytoskeletal proteins (28, 29) and is expressed equally on LPS plus IL-4 and LPS-stimulated B cell blasts (30). We have previously shown that CD44 localizes to the detergent insoluble membrane fraction (containing the cytoskeleton) in B cells stimulated with LPS + IL-4, but not when stimulated with LPS alone (16). We found that Dock10-deficient B cells spread similarly to control B cells in a wide range of IL-4 concentration and also at an early time point (Figures 4A,B; Figures S2A,B in Supplementary Material). Dock10-deficient B cells were able to polarize and migrate in response to IL-4 indistinguishable to WT B cells (Figures 4C,D). Moreover, the response to the chemokine CXCL12 was also similar in Dock10-deficient and WT B cells (Figure 4E). Another induced morphology change in B cells, which is not regulated by Cdc42, but by Rac1 and Rac2, is formation of large, dense aggregates (unpublished observations). We found that Dock10-deficient B cells formed large cellular aggregates in response to anti-CD40 + IL-4 and LPS + IL-4, as well as control B cells (Figure 4F). Together, this suggests that Dock10 is not required for IL-4-induced cytoskeletal changes in B cells.

### Decreased DNA Synthesis in Dock10 Knockout B Cells in Response to IL-4 and Anti-CD40 Stimulation

**Figure 5:**
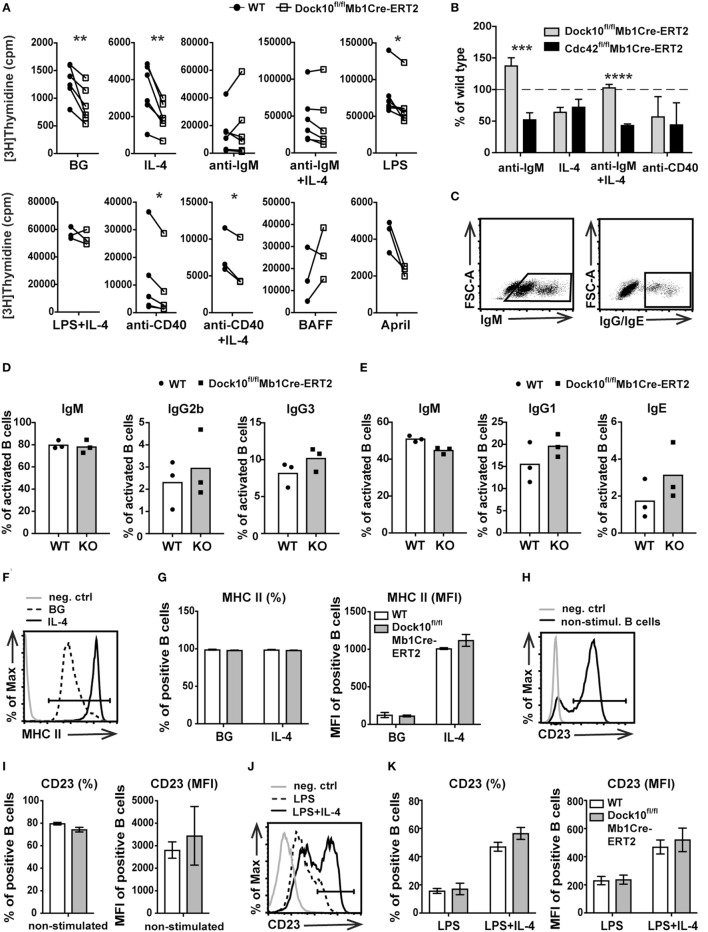
**Decreased DNA synthesis in Dock10 knockout B cells in response to interleukin-4 (IL-4) and anti-CD40 stimulation**. **(A)** [^3^H]thymidine uptake of spleen B cells from wildtype (WT) and Dock10^fl/fl^Mb1Cre-ERT2 mice, cultured for 2 days and [^3^H]thymidine added for the final 17 h. Each pair represents one experiment performed with triplicate wells. BG, background. Paired student’s *t*-test was performed (*p < 0.05, **p < 0.01). **(B)** [^3^H]thymidine uptake comparing Dock10^fl/ fl^Mb1Cre-ERT2 with Cdc42^fl/fl^Mb1Cre-ERT2 B cells. Percentage induction in cpm/culture compared to their respective WT controls. Bars represent mean values ± SD of triplicate wells. Unpaired student’s *t*-test was performed (***p < 0.001, ****p < 0.0001). **(C)** Representative graphs for the gating strategy of IgM or IgG/IgE B cells. **(D,E)** Ig class switching in spleen B cells from WT and Dock10^fl/fl^Mb1Cre-ERT2 mice. B cells were stimulated with LPS to measure intracellular IgG2b and IgG3 **(D)** or anti-CD40 + IL-4 to measure intracellular IgG1 and IgE **(E)**. **(F)** Representative flow cytometry histogram for MHC class II expression in B cells cultured without stimuli (BG) or with IL-4 for 24 h. **(G)** Percentage of MHC class II^+^ B cells (left) and the mean fluorescence intensity (MFI) of MHC class II^+^ cells (right). **(H)** Representative flow cytometry histogram for CD23 expression in non-stimulated (naïve) B cells. **(I)** Percentage of CD23^+^ B cells and MFI of CD23^+^ naïve B cells. **(J)** Representative flow cytometry histogram for CD23 expression in B cells cultured with LPS or LPS + IL-4 for 48 h. **(K)** Percentage of CD23^+^ B cells and MFI of CD23^+^ B cells stimulated with LPS and LPS + IL-4. **(D–K)** Mean and SD of three mice per group. Dock10^fl/fl^Mb1Cre-ERT2 mice were used. **(F,H,J)** neg. ctrl, negative control; BG, background.

To address how Dock10 in B cells may be important in signaling from different cell surface receptors, DNA synthesis was measured upon specific receptor stimulation. Dock10^fl/fl^Mb1Cre-ERT2 B cells showed a reduced response to IL-4, LPS, anti-CD40, and anti-CD40 + IL-4 when compared to control B cells (Figure 5A). On the contrary, the response to anti-IgM, with or without IL-4 as well as to BAFF or April was not significantly different from controls (Figure 5A). This raised the question whether Dock10-negative B cells were more prone to go into apoptosis in response to certain stimuli; however, Dock10 knockout B cells had similar proportion of apoptotic cells as control B cells (Figure S2C in Supplementary Material). Since Dock10 is a GEF for Cdc42, it was interesting to compare deletion of these molecules in the DNA synthesis response. The reduction of DNA synthesis in the response to IL-4 and anti-CD40 was similar in B cells lacking Cdc42 or Dock10 (Figure 5B). On the other hand, Cdc42 knockout B cells showed a reduced response to anti-IgM, whereas Dock10 knockout B cells did not (Figure 5B). Together, these data indicate that Dock10 acts as a GEF for Cdc42 in a receptor-selective manner. We next examined if the decreased proliferative response to LPS and anti-CD40 + IL-4 was associated with altered Ig class switching, known to be dependent on cell proliferation (31, 32). The production of IgM, IgG1, IgG2b, IgG3, or IgE responses were similar in Dock10 knockout and control B cells after *in vitro* activation with LPS or anti-CD40 + IL-4 (Figures 5C–E). We also tested other IL-4-dependent responses in B cells devoid of Dock10. Neither upregulation of MHC class II by IL-4 nor increased expression of CD23 by LPS + IL-4 were affected by the loss of Dock10 (Figures 5F–K).

### Deletion of Dock10 in B Cells Results in a Normal Immune Response to a Particulate Antigen, but a Decreased IgG Response to a Soluble TD Antigen

**Figure 6:**
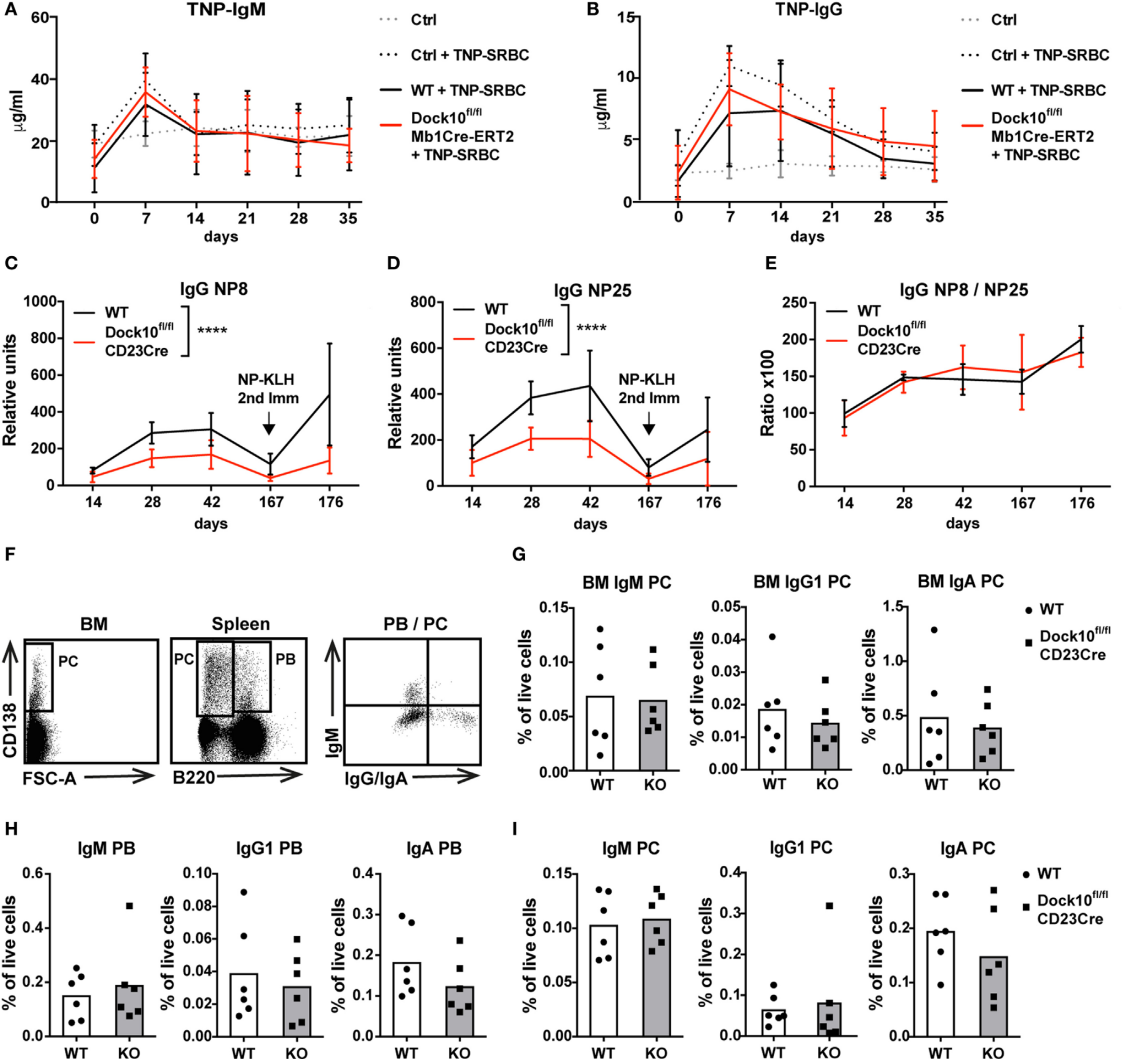
**Humoral immune response in mice with specific deletion of Dock10 in B cells**. **(A,B)** Mice were immunized with trinitrophenyl (TNP)-SRBC, and TNP-specific IgM **(A)** and IgG **(B)** on days 0–35 were examined in serum by ELISA. Dock10^fl/fl^Mb1Cre-ERT2 (Dock10^fl/fl^Mb1Cre) mice were given tamoxifen for 5 days prior to TNP-SRBC immunization, WT indicates Cre^−^ mice given tamoxifen, controls (Ctrl) indicate Cre^−^ mice that were not given tamoxifen. Immunized mice are indicated as +TNP-SRBC. Data in **(A,B)** represent Ctrl, *n* = 2, Ctrl + TNP-SRBC, *n* = 5, WT + TNP-SRBC, *n* = 7, Dock10^fl/fl^Mb1Cre-ERT2 + TNP-SRBC, *n* = 6. **(C–E)** Cre^−^ (WT) and Dock10^fl/fl^CD23Cre mice were immunized with NP-KLH, and NP-specific IgG were examined in serum by ELISA, on the indicated days. The mice got a challenge with antigen on day 167. The amount of serum antibodies was determined in ELISA using NP coupled to BSA with either 8 **(C)** or 25 **(D)** NP molecules per molecule of BSA. The ratio of the two responses is a measure of the relative affinity of antibodies **(E)**. Data in **(C–E)** represent WT, *n* = 2, Dock10^fl/ fl^CD23Cre, *n* = 2, WT + NP-KLH, *n* = 6, Dock10^fl/fl^CD23Cre + NP-KLH, *n* = 6. **(F)** Plasmablasts (PB) and plasma cells (PC) gating strategy in the bone marrow (CD138^+^) and spleen (CD138^+^B220^−^ PC and CD138^+^B220^+^ PB). **(G–I)** Flow cytometry analysis of IgM^+^, IgG1^+^, or IgA^+^ PB and PC from WT and Dock10^fl/ fl^CD23Cre mice immunized with NP-KLH and examined on day 176 in bone marrow **(G)** and spleen **(H,I)**. Data in **(F–I)** represent WT + NP-KLH, *n* = 6, Dock10^fl/fl^CD23Cre + NP-KLH, *n* = 6. ****p < 0.0001 using two way ANOVA for comparing WT versus Dock10^fl/fl^CD23Cre Ig titers at all time points.

To examine how B cell-specific deletion of Dock10 would influence the humoral immune response to a TD antigen, Dock10^fl/fl^Mb1Cre-ERT2 mice were given tamoxifen followed by immunization with the particulate antigen TNP-SRBC. WT and Dock10^fl/fl^Mb1Cre-ERT2 mice showed similar anti-TNP IgM and IgG responses up to 35 days after immunization (Figures 6A,B). In the Mb1Cre-ERT2 combination, there is a gradual reconstitution of WT B cells after a few weeks (4). In order to measure a prolonged immune response and a secondary challenge, Dock10^fl^ mice were bred together with CD23Cre mice. When Dock10^fl/fl^CD23Cre mice were immunized with NP-KLH, all mice responded with an NP-specific IgM response both after primary and secondary immunization, and the response was similar to controls (data not shown). The IgG response was significantly lower in Dock10^fl/fl^CD23Cre mice in the primary and might be different in the secondary response (Figures 6C,D). Interestingly, the affinity of the antibodies was similar in Dock10^fl/fl^CD23Cre and control mice as measured by the ratio of NP-specific IgG antibodies detected with haptenated carriers of low versus high density (Figure 6E). We next examined the generation of PB and PC. Dock10^fl/fl^CD23Cre mice showed normal generation of IgM^+^, IgG1^+^, and IgA^+^ PB and PC in bone marrow and spleen (Figures 6G–I). These data suggest that Dock10 KO mice formed GCs and had normal affinity maturation but produced less IgG antibodies despite having normal plasma cell generation in spleen and bone marrow. However, we have only investigated plasma cell levels at one time point, and thus, we cannot exclude that plasma cell development would be compromised at later times after immunization.

## Discussion

Dock10 is a GEF for Cdc42 and as such, one might expect that it would be important for B cell morphology changes, such as the spreading or adhesion response. This we showed not to be the case, perhaps implying redundancy between closely related GEFs in spreading responses or aggregation. As a whole, deletion of Dock10 had a relatively mild effect on B cells. The main observed phenotype of Dock10 deletion was a modest decrease in DNA synthesis in response to IL-4, LPS, and anti-CD40 + IL-4, but not to anti-IgM and a decreased IgG response to a TD soluble antigen, but with no effect on antibody affinity. This could be compared with the more severe phenotype observed when B cells lack Cdc42 (3, 4, 33). Our experiments suggest that another GEF, other than Dock10, is activating Cdc42 in response to B cell receptor signaling.

We have chosen to delete Dock10 in an inducible manner, to be able to distinguish the contribution of a deletion at early B cell differentiation stages from that of mature B cells. A consequence is that the phenotype would most likely appear milder than if deletion was made from early differentiation stages. We used a similar strategy in a recent paper to delete Cdc42 in B cells. We found that deletion of Cdc42 was maintained for 2 weeks after the last tamoxifen administration, but already after 3 weeks, Cdc42-sufficient B cells started to appear (4). Thus, by using the Mb1Cre-ERT2 combination, we can study effects up to a few weeks after onset of treatment. In the same paper, we also used CD23Cre expression to delete Cdc42 more permanently in mature B cells (4). By using a similar strategy, we were able to detect a mild deficiency in the IgG responses toward a soluble hapten-conjugated protein in Dock10-deficient mice.

Two recently published papers have reported data from Dock10 knockout mice. Matsuda et al. (34) deleted either Dock10 (Zizimin3) or Dock11 (Zizimin2) and found higher percentage of pre-pro-B cells in the bone marrow in the absence of either GEF. Furthermore, they found decreased percentage of marginal zone (MZ) B cells in the absence of Dock11 and a narrower MZ B cell region in mice lacking Dock10 or 11. In the other paper, García-Serna et al. (35) reported that deletion of Dock10 from early B cell stages leads to reduced numbers of B cells in the spleen. This resembles the effect of deletion of Rac1 and 2 in B cells, which leads to dramatically reduced numbers of B cells in the white pulp of the spleen (36). Thus, absence of Dock10 in immature B cells might lead to reduced ability of the cells to enter the spleen white pulp. García-Serna et al. (35) also found that CD23 was overexpressed in follicular B cells lacking Dock10. However, we did not find any significant difference in CD23 expression in Dock10-deficient B cells. García-Serna et al. (35) used the Dock10 mutant without deleting the neoR and the lacZ genes and that might lead to unwanted secondary effects. Furthermore, the effect on the B cell number might be indirect and due to Dock10 deletion in other cell types. In our study, we have deleted Dock10 in B cells only, and therefore the defects that we observe must be B cell intrinsic.

We found that deletion of Dock10 had no effect on the capacity of B cells to spread on antibody-coated surfaces. Burbage et al. (3) recently showed that BCR-dependent spreading on lipid monolayers was severely compromised in B cells devoid of Cdc42. Since we found that Dock10 respond normally to BCR signaling, it might be unlikely that Dock10 should play any role in BCR-dependent spreading. The two types of spreading are regulated quite differently. Whereas the BCR-dependent spreading is induced in a few minutes, the spreading induced by anti-CD40 + IL-4 is quite slow and requires several hours (16).

The absence of a large effect on the phenotype, when Dock10 was deleted in B cells, may be explained by compensatory mechanisms. Two other GEFs, Dock9 and Dock11, are closely related to Dock10. Dock9 was expressed at low levels in B cells, whereas Dock11 was expressed at higher levels than Dock10 in naïve B cells, but it was not further upregulated by stimuli. It is possible that Dock10 and Dock11 act in a redundant manner, as the study by Matsuda et al. (34) implies, and it would be possible to test this by deleting both these proteins simultaneously in B cells. On the other hand, other GEFs, such as Dock8 or Vav1–3, could have functions similar to Dock10 (37, 38). Alternatively, these GEFs could be involved in Cdc42-dependent signaling via the BCR.

Mice with Dock10-deficient B cells responded well to a TD particulate antigen, but less well to a soluble TD antigen. This is similar to the situation when B cells lack Cdc42 (4). A particulate antigen induces a much stronger response, perhaps because T cell help is more efficient or because SRBC induce a partly T cell independent response. It is possible that the reduced response to NP-KLH might be due to insufficient T cell help. However, this cannot be due to deficient expression of MHC class II or CD23 expression, since both expression and upregulation by IL-4 is intact in B cells lacking Dock10. Another possibility for the reduced response to NP-KLH might be decreased survival of Dock10-deficient B cells. The proliferation in response to the B cell survival factors BAFF and April was similar in Dock10-deficient and WT B cells. Together with normal apoptosis rate *in vitro* and normal affinity maturation as assessed by NP8/NP25 ratio upon NP-KLH immunization, our data suggest that Dock10-deficient B cells had capacity to undergo affinity maturation.

We observed a reduced DNA synthesis response in B cells, especially to IL-4 and anti-CD40 and also a significantly reduced response to a soluble TD antigen. However, the affinity of the IgG antibodies was not reduced. These two responses are most likely linked. A lower capacity of B cells to proliferate would be expected to have a reducing effect on the IgG response, since switching is linked to the capacity of cells to enter into S-phase and is occurring only after B cells have proliferated several times (31, 32). The puzzle is why lower proliferation capacity did not change the affinity of the response. Possibly, it could be related to the finding of Victora et al. (39), who screened for differences between GC compartments using a microarray profiling experiment and showed that the Dock10 gene is expressed at lower levels in the DZ as compared to that of the LZ. Using lacZ expression as a reporter of Dock10 promoter activity, we could here confirm lower Dock10 promoter activity in GC DZ B cells. It should be noted that by deleting Cdc42 in B cells, both the humoral immune response and the affinity maturation to soluble TD antigens is severely reduced (4).

We noticed an interesting difference in the proliferative responses in B cells lacking Cdc42 compared to that of Dock10: the level of reduction in response to IL-4 and anti-CD40 is similar, whereas Cdc42 deletion but not Dock10 deletion lead to a reduced response to anti-IgM. These data imply that different GEFs regulate the responses by associating with particular cell surface signaling receptors. Thus, another GEF than Dock10 might be responsible for activation of Cdc42 through the B cell receptor. Using a screen for genes that regulate cytoskeletal changes in B cells, we identified Dock10 that is specifically induced by IL-4 stimulation. However, specific deletion of Dock10 in B cells was associated with a mild phenotype suggesting that it is not absolutely required for B cell cytoskeletal changes, and that other related GEFs can compensate for the absence of Dock10 in B cells. To get a clearer picture, it would be interesting to study the effect of simultaneous deletion of Dock10 and Dock11 in B cells.

It is interesting to speculate what would happen if Dock10 was mutated in humans. Since it is more widely expressed than Dock11, it might result in a lethal phenotype (8). If it would be viable, our data, together with those of Matsuda et al. (34) and García-Serna et al. (35) indicate that it would result in immunodeficiency disease.

## Ethics Statement

All mouse strains were bred in specific pathogen-free conditions in the animal facility of the Department of Molecular Biosciences, the Wenner-Gren Institute, Stockholm University. All experiments using mice were approved by a local ethical committee on animal experiments.

## Author Contributions

NG, ES, and LW designed research and drafted the manuscript; NG, MH, MB, and ES performed the research; NG, MH, MB, ES, and LW analyzed the data and edited the manuscript.

## Conflict of Interest Statement

The authors declare that the research was conducted in the absence of any commercial or financial relationships that could be construed as a potential conflict of interest.

## Supplementary Material

Figure S1 **Microarray data**. Heatmaps showing gene expression profiling of mouse spleen B cells stimulated with anti-CD40 + IL-4, LPS, or non-stimulated spleen cells (left), and genes upregulated at least 10 times in B cells stimulated with anti-CD40 + IL-4 as compared to B cells stimulated with LPS (right). Genes are ranked as a fold change, data transformed by log2. IL-4, interleukin-4.

Figure S2 **(A)** Analysis of spreading in wildtype (WT) or Dock10^fl/fl^Mb1Cre-ERT2 B cells stimulated with LPS plus various concentrations of interleukin-4, as indicated in the graph. Cells were cultured for 20 h after transfer to anti-CD44-coated coverslips. Around 350–1,500 cells were counted per mouse per stimuli. The experiment was performed once. **(B)** Spreading analysis of WT or Dock10^fl/ fl^Mb1Cre-ERT2 B cells cultured for 2 h after transfer to anti-CD44-coated coverslips. Around 1,000 cells were counted per group. The experiment was performed once. **(C)** WT or Dock10^fl/fl^Mb1Cre-ERT2 B cells were stimulated with indicated stimuli for 3 days, and apoptosis was evaluated using annexin V and propidium iodide staining (apoptotic cells are positive for both markers). Data from one mouse per group are shown. The experiment was performed once with all stimuli but was performed one additional time with similar results after stimulation with anti-CD40.

Data Sheet S1 **Microarray data**. Sheet 1: genes upregulated by at least 2-fold in non-stimulated (BG) over LPS-stimulated cells; sheet 2: genes upregulated by at least 2-fold in non-stimulated (BG) over anti-CD40 + IL-4-stimulated (A) cells; sheet 3: genes upregulated by at least 2-fold in LPS-stimulated over anti-CD40 + IL-4-stimulated (A) cells; sheet 4: genes upregulated by at least 2-fold in anti-CD40 + IL-4-stimulated (A) over non-stimulated (BG) cells; sheet 5: genes upregulated by at least 2-fold in anti-CD40 + IL-4-stimulated (A) over LPS-stimulated cells; sheet 6: genes upregulated by at least 10-fold in anti-CD40 + IL-4-stimulated (A) over LPS-stimulated cells (*p* < 0.01 and *q* < 0.01). IL-4, interleukin-4.
