# Supplementary figures and images for: Deletion of Dock10 in B Cells Results in Normal Development but a Mild Deficiency upon In Vivo and In Vitro Stimulations
Source: Front Immunol. 2017 May 1;8:491. doi: 10.3389/fimmu.2017.00491 (PMC5410582; doi:10.3389/fimmu.2017.00491)

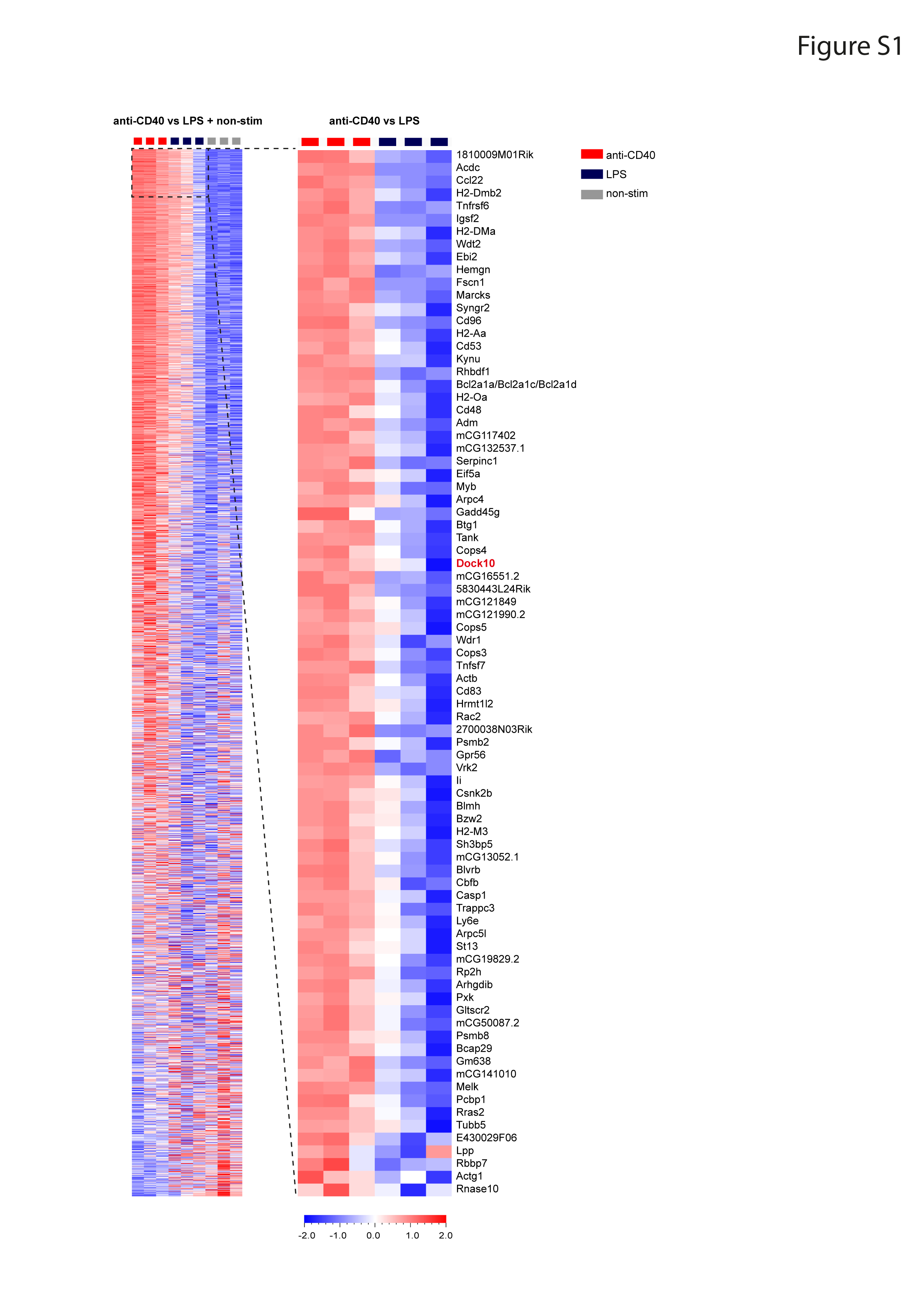

Supplement: Figure S1 — Microarray data. Heatmaps showing gene expression profiling of mouse spleen B cells stimulated with anti-CD40 + IL-4, LPS, or non-stimulated spleen cells (left), and genes upregulated at least 10 times in B cells stimulated with anti-CD40 + IL-4 as compared to B cells stimulated with LPS (right). Genes are ranked as a fold change, data transformed by log2. IL-4, interleukin-4. [file Image_1.TIF]

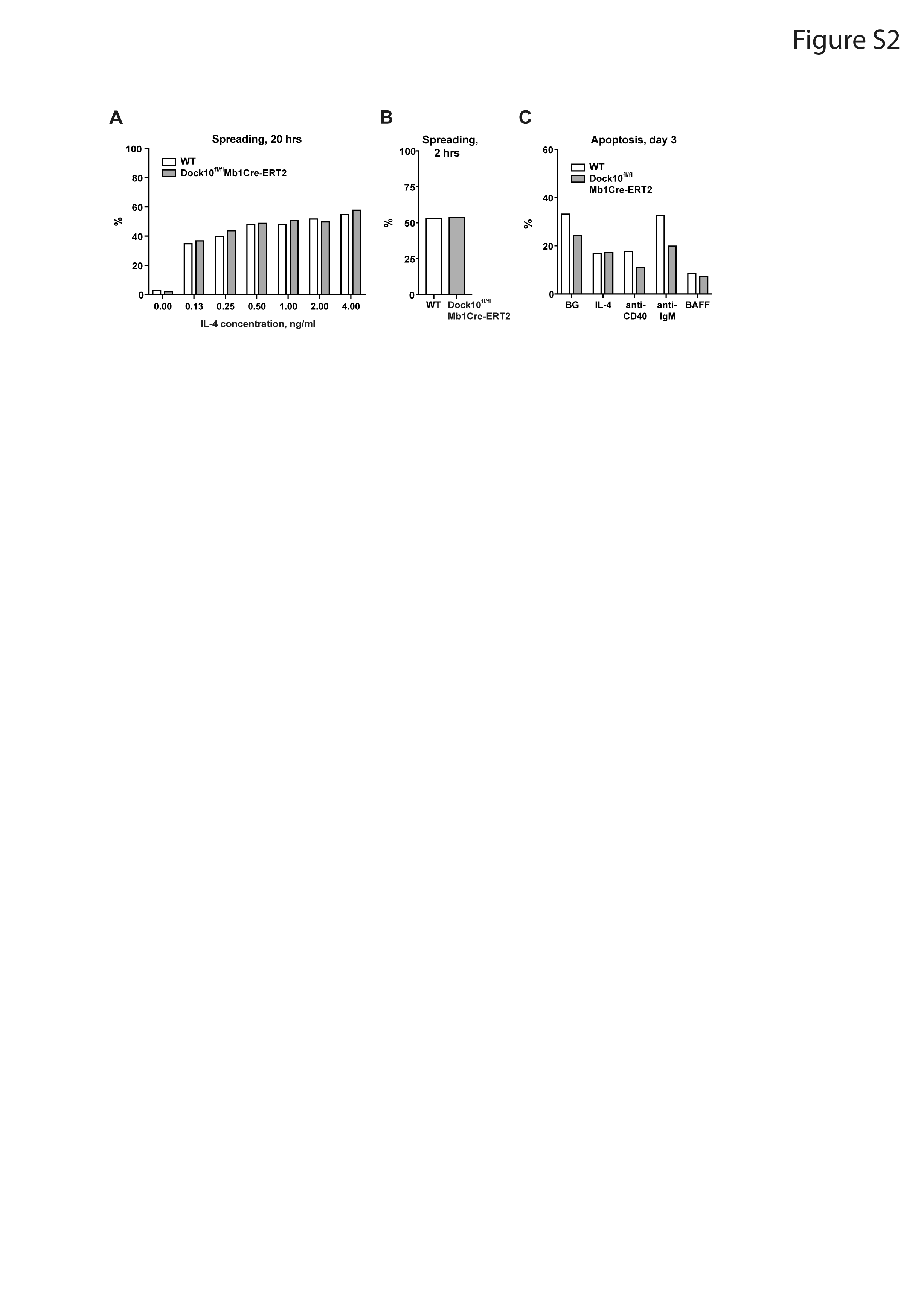

Supplement: Figure S2 — (A) Analysis of spreading in wildtype (WT) or Dock10fl/flMb1Cre-ERT2 B cells stimulated with LPS plus various concentrations of interleukin-4, as indicated in the graph. Cells were cultured for 20 h after transfer to anti-CD44-coated coverslips. Around 350–1,500 cells were counted per mouse per stimuli. The experiment was performed once. (B) Spreading analysis of WT or Dock10fl/ flMb1Cre-ERT2 B cells cultured for 2 h after transfer to anti-CD44-coated coverslips. Around 1,000 cells were counted per group. The experiment was performed once. (C) WT or Dock10fl/flMb1Cre-ERT2 B cells were stimulated with indicated stimuli for 3 days, and apoptosis was evaluated using annexin V and propidium iodide staining (apoptotic cells are positive for both markers). Data from one mouse per group are shown. The experiment was performed once with all stimuli but was performed one additional time with similar results after stimulation with anti-CD40. [file Image_2.TIF]
